# Dimensionality of instructional quality in physical education. Obtaining students’ perceptions using bifactor exploratory structural equation modeling and multilevel confirmatory factor analysis

**DOI:** 10.3389/fpsyg.2024.1370407

**Published:** 2024-08-19

**Authors:** Felix Kruse, Sonja Büchel, Christian Brühwiler

**Affiliations:** ^1^Institute of Physical Education, Sports and Health, St. Gallen University of Teacher Education, St. Gallen, Switzerland; ^2^Institute of Education and Professional Studies, St. Gallen University of Teacher Education, St. Gallen, Switzerland; ^3^Vice-President’s Office for Research & Development, St. Gallen University of Teacher Education, St. Gallen, Switzerland

**Keywords:** instructional quality, students’ perceptions, physical education, MCFA, ESEM, B-ESEM, teaching quality

## Abstract

**Background:**

In research on instructional quality, the generic model of the three basic dimensions is an established framework, which postulates that the three dimensions of classroom management, student support and cognitive activation represent quality characteristics of instruction that can be generalized across subjects. However, there are hardly any studies that examine if the three basic dimensions model could represent a suitable approach to measure instructional quality in physical education. Based on an extended model of the basic dimensions, a measurement model of instructional quality for physical education is presented, which integrates different theoretical approaches from the fields of educational and psychological research as well as different subfields of sports science in order to test the factorial structure of the corresponding measurement model.

**Methods:**

1,047 students from 72 seventh to ninth grade classes from different German-speaking Swiss cantons participated in the study. The conceptualization of the instrument is based on a hybrid approach that integrates generic and subject-specific characteristics. The simultaneous analysis at the individual and class level using MCFA was supplemented by more complex methodological techniques within the relatively new B-ESEM framework at the individual level.

**Results:**

The postulated five-factor structure was initially tested using ICM-CFA and showed a good model fit (e.g., χ^2^/df = 2.32, RMSEA = 0.03, CFI = 0.97, TLI = 0.97, SRMR = 0.04). MCFA revealed a differential factorial structure at both levels of analysis with five factors at the individual level and four factors at the class level (e.g., χ^2^/df = 2.23, RMSEA = 0.03, CFI = 0.96, TLI = 0.96, SRMR within = 0.04, SRMR between = 0.10). ESEM and B-ESEM outperformed the ICM-CFA and showed an excellent model fit (B-ESEM: χ^2^/df = 1.19, RMSEA = 0.01, CFI = 1.00, TLI = 1.00, SRMR = 0.01). Inter-factor correlations and factor loadings are largely in line with expectations, indicating arguments for construct validity.

**Discussion:**

The study represents a substantial contribution in linking physical education and the generic research on instructional quality. Overall, strong arguments for the factorial structure of the measurement model were demonstrated. The study can be interpreted as a first step in a multi-step procedure in terms of further validity arguments.

## 1 Introduction

Instructional quality has proven to be one of the strongest predictors of educational outcomes like achievement and motivation (e.g., Seidel and Shavelson, 2007). Although there is a consensus on the multi-dimensionality of instructional quality (e.g., Klieme et al., 2001; Kyriakides and Creemers, 2008), current contributions deal with the differentiation of various dimensions, especially regarding different subjects (Praetorius et al., 2020a). Even if theoretical background and measurement diverge strongly (Praetorius et al., 2018; Bijlsma et al., 2021), the consensus may be that at least three dimensions of instructional quality can be distinguished (Pianta and Hamre, 2009; Klieme, 2013). Although this conception is particularly appealing for its parsimony, recent contributions confront the model with the question of whether this threefold structure is comprehensive enough (Praetorius and Charalambous, 2018; Kleickmann et al., 2020). Whereas the majority of empirical evidence can be found in mathematics and science education (Praetorius et al., 2020a,b,c), there is a lack of empirical evidence for physical education (PE). For PE, which differs from predominantly cognitive subjects in various aspects (e.g., the relevance of motor functions), the question arises to what extent generic conceptualizations can be transferred and to what extent they should be adapted and supplemented in a subject-specific way. However, in connecting PE to the generic research on instructional quality, it seems to be a suitable approach to use the evidence already available from other subjects to the best potential. Accordingly, the present study investigates the dimensionality of instructional quality in PE using the combination of generic and subject-specific aspects. Since we postulate that interindividual differences hold special significance in PE, in addition to the simultaneous analysis at the individual and class level using multilevel confirmatory factor analysis (MCFA), relatively new promising methodological approaches are applied at the individual level using a combination of Bifactor Modeling and Exploratory Structural Equation Modeling (B-ESEM).

### 1.1 Students’ perceptions of instructional quality

Ensuring that scientific quality criteria are met is a central issue in research on instructional quality (Göllner et al., 2021). Together with external observations, students’ perceptions are one of the central data sources for assessing instructional quality. While external observations provide a higher degree of objectivity and can make evidence-based assessments (assuming observers have been well trained), to be truly reliable, these must be conducted by several observers over several lessons (Praetorius et al., 2014). Thus, external observation can generally be described as time-consuming and relatively expensive. While external observation often tend to be considered as the gold standard, students’ perceptions have been shown to have the potential to provide reliable and valid information for the study of instructional quality (Fauth et al., 2014; Kane et al., 2014). Students contain a long-term experience with the teacher, are able to compare teachers inter-individually and being highly economical to conduct. Moreover, the large number of observers could improve the reliability at class level (e.g., Kane and Staiger, 2012; Fauth et al., 2020). Furthermore, the use of student perceptions provides the opportunity to examine not only data at the class level, but also at the individual level, so that the information regarding differences within classes can be examined. Accordingly, there are additional possibilities for a deeper insight into the data, which can be used to address research questions that focus on inter-individual differences. However, using students’ perceptions of their instructional quality (SPIQ) in research can be described as a complex endeavor. Researchers concerned with the measurement of SPIQ are confronted with the question of what has to be taken into account to ensure that they represent reliable and valid measurements. That is, for example, the interpretation of the items by the recipients in relation to the intention of the test constructor (Karabenick et al., 2007), the issue of low agreement with other data sources (Kunter et al., 2007; Wagner et al., 2016), the idiosyncratic nature of students’ ratings (Göllner et al., 2018, 2021), the generalizability of domain-independent assessments or the high inter-factor correlations of theoretical distinct instructional quality dimensions (Röhl and Rollett, 2021). With regard to the last point, it can be stated that although in principle there is evidence for the factorial validity of SPIQ, studies report very high inter-factor correlations of the theoretically divergent dimensions. For example, Krammer et al. (2019) report an inter-factor correlation of up to 0.95 at the individual level and 0.93 at the class level, Wagner et al. (2013) report values of up to 0.74 at the individual and 0.94 at the class level, and Wisniewski et al. (2020) report values of up to 0.89 at the individual and 0.93 at the class level. Some researchers, looking at the specifically used items in the different studies, tried to explain some of the mentioned challenges in the use of SPIQ. For example, regarding the reference of the item in the context of the low agreement of different data sources (Fauth et al., 2020) or regarding halo effects as a possible explanation for high inter-factor correlations (Röhl and Rollett, 2021).

### 1.2 Dimensionality of instructional quality

Concerning the measurement of instructional quality, a variety of approaches exist, whereby the question of parsimony as well as comprehensiveness arises. Certainly, the complexity of teaching must be reduced, so that it becomes manageable in some way. On the other side, the important teaching aspects for the achievement of educational goals should be incorporated (Praetorius et al., 2020c). A prominent model of instructional quality in the context of condensing key instructional aspects as parsimoniously as possible have been developed by Klieme et al. (2001). The model includes the three basic dimensions (TBD) of *classroom management*, *supportive climate* and *cognitive activation*. *Classroom management* can be described as a prominent construct in educational research and includes the strengthening of desirable student behaviors by, for example, communicate clear rules, and preventing undesirable student behavior, e.g., by monitoring or designing transitions (e.g., Kounin, 1970; Hochweber et al., 2014). These behaviors may manifest in low-disruptive classroom environments, which are like to promote the transition of potential learning time into real learning time (Kuger et al., 2016). Provided that it is used, classroom management is considered to be central to student learning success and may foster student motivation as well (Rakoczy et al., 2007; Seidel and Shavelson, 2007). *Student support* is characterized by the student-teacher relationship and includes aspects such as caring behavior, support for autonomy or a positive approach to mistakes. Because of the emotional character of these factors, effects on social–emotional outcomes in particular are assumed (Fauth et al., 2014; Praetorius et al., 2018). Finally *cognitive activation* is based on constructivists views of learning and contains addressing students’ prior knowledge, challenging tasks, stimulation of cognitive conflicts or the engagement of students in higher-order thinking processes (Lipowsky et al., 2009; Baumert et al., 2010).

The model of the three basic dimensions is particularly appealing because of its theoretical foundation as well as the parsimony of the model (Praetorius et al., 2020b). In recent times, however, the question has arisen repeatedly as to what extent the three dimensions are comprehensive enough, or whether it would make sense to add further dimensions. In their review, Praetorius et al. (2018) found that only half of the previous findings were consistent with the model assumptions and accordingly suggest further development of the three basic dimensions model. Kleickmann et al. (2020) proposed a for PE interesting addition of a fourth dimension, namely cognitive support. Cognitive support is based on theories from cognitive psychology as well as social constructivist theories. Drawing particularly on cognitive load theory and the role of scaffolding in complex learning situations, cognitive support aims to reduce complexity and corresponding cognitive demands. Given this background, Puntambekar and Hubscher (2005) differentiate between an original and an evolved or current notion of scaffolding. Kleickmann et al. (2020), following this literature, distinguish between adjusted support, which particularly involves the interaction of teachers and students by means of explaining, highlighting, and informative feedback, and blanked support, which relates more to a collective level by establishing clarity of goals or a clear structure. Since cognitive support can be understood as a significant instructional dimension, it would be surprising if no integration had taken place in the model of the three basic dimensions. Due to the heterogeneity of operationalizations of the model of the three basic dimensions (Praetorius et al., 2018), Kleickmann et al. (2020) compile different types of integration of cognitive support in previous work on the three basic dimensions, which can be divided into four types: First type contains no or only rudimentary consideration in the three basic dimensions. If considered, then as part of student support (examples include Fauth et al., 2014; Decristan et al., 2015). The second type provides a more comprehensive integration of cognitive support into student support. In this case, student support is divided into a cognitive and a motivational component, with the former involving the reduction of cognitive demands and the latter involving social relatedness and autonomy support (examples include Kunter and Voss, 2013; Hochweber and Vieluf, 2018). In the third type, cognitive support is integrated as part of classroom management; especially as lesson clarity or structure (examples include Klieme et al., 2001; Taut and Rakoczy, 2016). Finally, the fourth type subsumes cognitive support under the basic dimension of cognitive activation. The Classroom Assessment Scoring System (CLASS; Pianta and Hamre, 2009) can be mentioned as an example, in which aspects such as the quality of feedback or the clear presentation of content (cognitive support) as well as the promotion of higher-order thinking (cognitive activation) are integrated. The empirical analysis of the postulated four-factor structure of classroom management, motivational support, cognitive support, and cognitive activation using SPIQ for science education shows an adequate model fit, representing the favored model over the alternative models (types 2–4). However, a closer look at the items of the study by Kleickmann et al. (2020) shows that the operationalization of cognitive support includes in particular the reduction of complexity in the sense of the occurrence of and help with comprehension problems as well as the clarity of goals. Therefore, further aspects such as modeling, explaining, or highlighting or the reduction of task difficulty are not or only marginally reflected. Due to this sparse operationalization as well as the theoretical differentiation of adjusted and blanked support, the question arises to what extent these two could also represent independent factors, representing a five-factor structure. Considering other models of instructional quality, such a division can also be observed, for example in the teaCH model in which aspects of adjusted and blanked support are largely modeled separately (e.g., Kane and Staiger, 2012; Wisniewski et al., 2020). These two components of cognitive support appear to be a potent extension of the model of the three basic dimensions in order to integrate significant PE-specific aspects of instructional quality.

### 1.3 Instructional quality in physical education: what to adapt, what to extend?

#### 1.3.1 Characteristics of physical education and corresponding objectives

Existing instruments of instructional quality vary widely in the scope and selection of relevant dimensions (e.g., Charalambous and Praetorius, 2018; Bijlsma et al., 2021). This is not least the case since it can be described as difficult to neither under-represent a construct nor to include aspects that are less relevant to the target criterion (AERA et al., 2014). Correspondingly, the conceptualization of instructional quality should be carried out in terms of the intended educational goals and in relation to the scope of the respective study. Even if there is no international consensus, the goals of PE can be described as at least partly different to other subjects. In this context, cognitive outcomes are less relevant to most other subject matters. Instead, PE provides a unique contribution to the education of students in the context of motor competence (e.g., Rink, 2014). However, it seems important to emphasize that PE does not necessarily aim at peak performance of the students’ motor competencies but rather target basic motor competencies that could be shown to be significant prerequisites for physical activity (PA) (McLennan and Thompson, 2015; Lopes et al., 2021). In this context, PE has great potential to promote PA not only during school hours, but also to acquire the necessary motor competencies and motivation to be physically active outside of school (e.g., Jaakkola et al., 2017). Given that physically inactive children are more likely to become physically inactive adults (Telama, 2009), in line with McLennan and Thompson (2015), quality PE has great potential to be the foundation of lifelong participation in PA, which in turn, can be considered a key health variable associated with multiple physical health benefits, such as cardiovascular and metabolic health, as well as mental and cognitive health benefits (Janssen and LeBlanc, 2010; Poitras et al., 2016; Biddle et al., 2019; Whooten et al., 2019). Besides motor competencies, motivational variables play a pivotal role in PE. Especially a lack of enjoyment and low perceptions of physical competence can be identified as particular important influencing factors regarding PA (Sallis et al., 2000; Babic et al., 2014; Crane and Temple, 2015; Jaakkola et al., 2017). Therefore, as one key goal of PE, students should be offered a motivationally and emotionally supportive environment in which they can adequately develop motor competencies to stay physically active across the lifespan. It must be noted, however, that even though it is assumed that enjoyable experiences in PE can create a positive emotional state that may encourage participation in PA during leisure time, which is also supported by the trans-contextual model (Hagger et al., 2003), for which there is some empirical evidence (Hagger et al., 2009), it does not fully account for the affective responses that may partly explain the relationship between PE motivation and PA participation. It is noteworthy that enjoyment in PE accounted for only 10–15% of PA participation, suggesting that PA participation is also influenced by various other factors (Sallis et al., 2000) in addition to enjoyment in school PE.

Considering the mentioned objectives of PE, the following section summarizes relevant evidence on instructional variables for PE in the context of the generic model of the three basic dimensions and the highlighted outcomes of motivation, perceived competence and motor competencies. However, as the number of variables under consideration is large and the contexts and settings of studies varies widely, the present study can only represent a selection of potentially significant factors.

#### 1.3.2 Motivational psychology approaches

Motivational processes play a critical role in physical education (PE) by shaping how students engage with and benefit from the instructional environment. Integrating principles from motivational psychology can significantly enhance instructional quality and student outcomes. Drawing on self-determination theory (Deci and Ryan, 2000), a key element in creating an effective PE environment is the fulfillment of the basic psychological needs of autonomy, competence, and relatedness. The support of basic psychological needs can be seen as typical aspects of an instructional quality understanding and is even part of the theoretical foundation of the same (for TBD, see Praetorius et al., 2018). Need supportive practices are positively associated with need satisfaction, more autonomous SDT types of regulation as well as adaptive outcomes (e.g., enjoyment and physical activity intentions), whereby teachers have more potential to influence students’ autonomy and competence compared to students’ relatedness (Vasconcellos et al., 2020). Furthermore, the relatedness between peers seems to play an important role in the development of intrinsic motivation (Vasconcellos et al., 2020; Kruse et al., 2024). In addition to fulfilling these basic psychological needs, establishing a mastery-oriented motivational climate is essential for fostering students’ intrinsic motivation and long-term engagement in physical activities. This climate emphasizes personal improvement, effort, and learning rather than competition and comparison with others. By focusing on personal goals and self-improvement, students are encouraged to view challenges as opportunities for growth, which fosters intrinsic motivation (Ames, 1992; Soini et al., 2014), which is in line research on teachers’ individual reference norm orientation (e.g., Dickhäuser et al., 2017). Incorporating these motivational elements into PE is not without its challenges. The physical and often public nature of PE activities can make students feel vulnerable, leading to heightened emotional responses such as anxiety or embarrassment. Teachers must skillfully manage these emotional dynamics to maintain a positive and productive learning environment (Gerlach et al., 2007; Sabiston et al., 2014). An emotionally and motivationally supportive classroom environment may help to mitigate negative emotions, encouraging students to participate more actively and confidently. In addition, positive feedback, which focuses on successful performance and provides constructive guidance, significantly boosts students’ sense of competence and motivation. For example, Badami et al. (2011) found that feedback following successful trials enhances intrinsic motivation more effectively than feedback after unsuccessful trials. This aligns with findings by Saemi et al. (2012), who demonstrated that learners receiving feedback on their successful attempts exhibited higher levels of perceived competence and intrinsic motivation compared to those who received feedback on their errors.

#### 1.3.3 Classroom management

Regarding the specifics of classroom management in PE, many authors describe classroom management as more challenging than in other subjects, referring particularly to the difficult acoustics in the gym, the lack of pre-structured space compared to a classroom, the changing teaching locations, and the safety aspect (Chepyator-Thomson and Liu, 2003; Cothran and Kulinna, 2014; Baumgartner et al., 2020). Empirical evidence is primarily found in the area of measurement instruments of classroom management (Baumgartner et al., 2020, 2023) or oftentimes disruptive behavior (Krech et al., 2010) as well as regarding the prerequisites for disciplined behavior (Claver et al., 2020), whereas the effects of classroom management on student outcomes are more implicitly assumed. However, Bevans et al. (2010) highlight the impact of classroom management on student physical activity levels during PE.

#### 1.3.4 Cognitive-motor support and cognitive-motor activation

While subject-specific additions and adaptations are less pronounced in classroom management and student support, they are considered most challenging for cognitive activation (Schlesinger et al., 2018) and, in context of this study, likewise for cognitive support. In contrast to the more cognitive shaped subjects, the connection between cognition and motor learning is of particular importance for PE. Accordingly, it is important to identify, to what extent these dimensions must be adapted to take this difference into account. From a neuroscience perspective, the interconnection of cognition and motor function can be underpinned by internal model theory. It posits that the process of motor control is closely linked to the construction and updating of mental models that describe the relationship between actions and sensory feedback (Wolpert and Flanagan, 2016). These mental models serve as a frame of reference for monitoring and correcting movements and allow us to anticipate the effects of changes in the environment or body on our movements. The internal model is built through motor experience and can be updated through feedback from the environment (Shadmehr and Krakauer, 2008). It consists of various components that control the dynamics and stability of movements, such as prediction models based on sensory information and correction models that calculate and compensate for errors between actual and desired movement. By building and improving internal models through motor experiences, learners can optimize their movement skills and respond more quickly to new situations and environments. Internal model theory understands motor learning as an active process, as our movements influence our sensory information (Wolpert et al., 2011). It appears obvious that the generic dimensions of cognitive activation and cognitive support are therefore in no way to be understood without a motor complement. Therefore, in the following, the two dimensions are consequently termed cognitive-motor activation and cognitive-motor support. Even though a co-constructive learning situation is clearly not the scope of the internal model theory, it seems reasonable that by, e.g., gradually introducing students to more complex movements and providing feedback teachers can continuously improve and refine their internal models of movements.

For blanket cognitive-motor support, in line with a constraint-based perspective of motor learning (Renshaw et al., 2010), it can be assumed that by constraining the dynamic interplay of the performer, the task, and the environment through the guidance of the teacher, individualized support becomes facilitated. The emphasis on the role of teacher guidance of students in complex learning environments is congruent with theories of cognitive psychology, especially cognitive load theory, which aim to reduce complexity in learning situations (e.g., Kirschner et al., 2006). While scaffolding is relevant for both subcomponents, but especially for blanked support, teacher feedback is particularly relevant for adjusted support. In this context, feedback is one on the most researched instructional aspects in PE. However, the relevant literature on motor learning and motor control refers to augmented feedback, that is the information provided by sources outside the body, like visual, auditory and multimodal feedback (Moinuddin et al., 2021)—a term that does not appear in research on generic instructional quality but is necessary in PE because of its distinction from sensory feedback (Cole and Sedgwick, 1992). As in other subjects, (augmented) feedback can be considered an essential instructional factor for quality PE. Empirical findings show that augmented feedback can promote motivation and perceived competence (Mouratidis et al., 2008) as well as motor learning (Zhou et al., 2021). In the literature on motor learning, augmented feedback can be divided into information about the result of the movement (knowledge of result) and about the quality of the movement (knowledge of performance) (Lauber and Keller, 2014) as well as with regard to the temporal dimension in giving feedback immediately (concurrent feedback) and giving feedback after the execution of the movement (terminal feedback) (e.g., Moinuddin et al., 2021). It seems reasonable that the relevance for PE of the different types differs especially in relation to the corresponding sport. In long jump, for example, knowledge of result can be obtained largely without feedback from the teacher, whereas for esthetic criteria in gymnastics or dancing, external feedback on the result of the movements can be perceived as very significant. Verbally given feedback about knowledge of performance is most common in PE and can be divided in a prescriptive and a descriptive component (Schmidt et al., 2018; Petancevski et al., 2022). The two components aim at advising the learner to improve the movement as well as correcting movement errors. While positive effects of the prescriptive component on motor performance have been demonstrated in an adult population, Petancevski et al. (2022) emphasize the large positive effects of a combination of both components in their recently published systematic review. Accordingly, both components appear to be potentially relevant instructional aspects for PE. Furthermore, systematic reviews have also addressed the question of which subtypes (e.g., verbal, visual; informative, corrective, evaluative) of feedback support motor learning most strongly under certain conditions in PE (e.g., task complexity; skill level). The findings can be described as partially inconsistent, whereby in the case of verbal feedback, only corrective feedback proved to be effective for motor learning. However, it should be emphasized that the formats and contents of the underlying studies differ considerably (Zhou et al., 2021; Han et al., 2022).

With regard to cognitive-motor activation, limitations can be identified in the transfer to PE with regard to the concept of higher-order thinking. This circumstance is also relevant to adjusted cognitive-motor support, whereby research on focus of attention is particularly relevant. Research on focus of attention is mainly concerned with the question of whether an external focus of attention (focusing on movement effects) or an internal focus of attention (focusing on movement form) is more conducive to movement learning. The large majority of research indicates that an external focus of attention in different contexts, such as task type or age groups, leads to improved outcomes of both movement effectiveness (e.g., accuracy, balance) and movement efficiency (e.g., muscle activity, cardiovascular responses) (Wulf, 2013). Overall, an external focus of attention appears to be a beneficial condition for optimal motor learning. The assumption is that a learner’s focus on the process of movement execution disrupts the automatic processes that control movement, resulting in lower motor performance. However, addressing this possible limitation of transferability seems to be a worthwhile endeavor in an empirical investigation.

Nesbitt et al. (2021) identified additional factors that are relevant within the extended model of the three basic dimensions in question, namely, developmentally sequenced activities, task-relevant cues, and emphasis on instruction and feedback on an individual basis. Furthermore, regarding the transfer of TBD to PE, a first instrument was presented by Herrmann (2019), which was complemented by a subject-specific motor activation dimension, based on the action-theoretical perspective of Niederkofler and Amesberger (2016). In doing so, Herrmann constructed and adapted items for PE in a subject-specific manner. However, a confirmatory analysis of the dimensionality of the model as well as further analysis has not yet been carried out so far.

#### 1.3.5 The appropriate level of analysis

Combining the constructivist view of TBD and motor research on learning as an active, individual process it can be assumed that the degree of cognitive-motor activation and cognitive-motor support varies greatly between individuals and manifests itself differently in the context of individual learning conditions. That is, a specific movement task may be strongly cognitively activating for one student, whereas it may be less cognitively activating for another student. Drawing on the social constructivist assumption of the zone of proximal development, it can be assumed for cognitive-motor activation that students must rather feel individually challenged in order to stimulate learning processes (Vygotsky and Cole, 1978; Rieser and Decristan, 2023). For aspects of adjusted cognitive-motor support, it seems reasonable that this focus on individual level might also apply. For instance, it can be assumed that a substantial part of the teacher’s feedback is individualized and does not take place at the class level. According to these assumptions, it seems reasonable that cognitive-motor activation as well as adjusted cognitive-motor support should be more strongly conceptualized as individual-level constructs instead of classroom-level constructs (Marsh et al., 2012; Rieser and Decristan, 2023). In this respect, deviations of individuals from respective class means as inter-individual differences should considered as important indicators for adequate support of the teacher (Göllner et al., 2018). Here, the specific construct should be considered on a customized basis: If, for example, items were asked about disruptive behavior in the classroom, the degree of interindividual differences would presumably be significantly lower than would be the case for items about individual augmented feedback in the context of cognitive-motor support. Indeed, Wagner et al. (2016) were able to show high consistency in student ratings for constructs such as classroom management or goal clarity, whereas constructs such as autonomy support, with a stronger individual emphasis, showed low consistency. Identifying the appropriate level of analysis is an important issue. Lüdtke et al. (2009) clearly emphasize that the appropriate level of analysis depends on the specific research question. Taking into account that motivational processes play a significant role in PE and that these usually have low intra-class correlations, the importance of the individual level can be emphasized (Kunter et al., 2007; Lazarides and Ittel, 2012). Moreover, we would like to point out another possible condition for the choice of the level of analysis in PE. In contrast to other subjects, it can be assumed that inter-individual differences are particularly formative. PE is shaped by the reciprocal relationship of extracurricular and school sport practice. The former obviously has a great influence on the students’ learning performance as well as on the overall performance heterogeneity within the class. Furthermore, extracurricular sports are largely organized in individual sports (e.g., soccer, dancing, and gymnastics), which exacerbates performance differences in PE. Taking, for instance, the subject of mathematics, there will be only a fraction of learners who participate in a mathematical recreational activity. If they do, it will probably be mostly not with strong limitation to a subfield of mathematics. Inter-individual differences thus seem to play a pivotal role in PE, which are not exclusively related to the achievement level but also to motivational aspects (e.g., strong interest in basketball, weak interest in dancing). In connection with the above-mentioned social constructivist views, the individual level for the dimension of adjusted cognitive-motor support, cognitive-motor activation and motivational-emotional support can be considered as particularly relevant. Students must be individually cognitively challenged and receive support related to their learning level, which should ultimately manifest itself in an improved motor learning performance as well as in an increase in intrinsic motivation of the students. These assumptions can also be supported by models of motor learning. The popular three-stages view (Fitts and Posner, 1967) can be used as an example. Overall, the interplay between cognition and motor systems occurs at different levels of movement learning. At the beginning of the learning process (cognitive stage), it is necessary for the learner to develop an understanding of the movement to be learned. Learners performing a movement task for the first time are confronted with the question of what actions, on an initially rather granular level, need to be performed in order to achieve the intended goal. This requires cognitive processing of the movement requirements and planning of the movement sequences. Learners attempt to develop appropriate strategies to realize adequate movement execution. This stage is likely to be characterized by a particularly high level of cognitive activity, supported by verbal feedback in particular (Fitts and Posner, 1967; Schmidt et al., 2018). Furthermore, it is characterized by a high increase and a high inconsistency in performance. Therefore, the cognitive stage is the most appropriate for teachers to support the learning process, e.g., through structuring and feedback. Once the understanding of the movement is in place, the motor implementation of the movement begins (fixation stage). Performance improvement is mostly gradual, less inconsistent and can persist over a long time. The focus is now less on the question of relevant movement patterns but more on the question of how movement execution can be optimized. The importance of instruction decreases, whereas the importance of sensory feedback increases. These two of the three stages already illustrate very well the importance of individual feedback, depending on the performance level of the student (Edwards, 2010; Braun et al., 2017). Considering the importance of basic motor competencies in PE, it quickly becomes apparent how relevant the cognitive phase is, since here the aim is not to perform at a higher level, but to refer to the participatory idea of PE (see also the ability to act; Gogoll, 2013).

### 1.4 Promising advanced methodological techniques

High inter-factor correlations between dimensions of instructional quality raise the question of whether students’ perceptions can adequately distinguish between them, respectively, whether the different factors are strictly distinct (Scherer et al., 2016; Röhl and Rollett, 2021). In this context, the typical investigation of multidimensional instruments in psychological and educational research is based on confirmatory factor analysis (CFA). However, despite the various important contributions (Marsh et al., 2014), CFA is based on the Independent Cluster Model (ICM), in which cross-loadings between items and non-target factors are fixed to be exactly zero (e.g., Howard et al., 2016). Regarding the mentioned high inter-factor correlations of instructional quality dimensions, from a measurement perspective, not taking into account that items potentially belong to one or more other factors, can also be reflected in inflated inter-factor correlations as well as poor goodness-of-fit. Taking this into consideration, more flexible models such as exploratory structural equation modeling (ESEM) have recently been introduced, which overcome the unrealistic ICM assumptions and, conversely, represent a more realistic modeling approach. As its name already indicates, there are similarities between ESEM and conventional exploratory factor analysis (EFA) in that cross-loadings between items and all factors are allowed. However, ESEM differs from EFA in that it incorporates features of structural equation modeling and therefore allows the evaluation of model fit indices, the assessment of measurement error, or the testing of measurement invariance. ESEM can thus integrate the best of both approaches, the EFA and the ICM-CFA, in one model. The lack of consideration of cross-loadings in the context of CFA can bring disadvantages, which are particularly important for constructs like instructional quality, where it is assumed that the different dimensions have conceptual overlaps. The examples of heterogeneous incorporation of cognitive support into different dimensions (see section 1.2) can be cited as a suitable example in this context. Specifically, ignoring cross-loadings can lead to biased results regarding inflated inter-factor correlations of factors as well as a reduction in goodness-of-fit indices (e.g., Marsh et al., 2020; Alamer, 2022). Accordingly, the high inter-factor correlations of the factors in the field of instructional quality would not necessarily have to be regarded as weak discriminant validity but may also indicate the disadvantages of the ICM assumptions. Further problems occur in the context of typical, subsequent adjustments in the context of ICM-CFA as a result of poor model fit (e.g., allowing measurement errors to correlate or removing items; Alamer, 2022). Removing items in this context can be seen as particularly difficult in the context of parsimonious modeling (such as the model of instructional quality in question). Especially, when the number of items measuring a construct is limited, each item contains important information about the construct, so that removing the item would distort the representation of the construct (Hair et al., 2019). Considering the advantageous features of ESEM, an application in the field of instructional quality research seems to be a promising approach.

The assessment of hierarchically ordered constructs, in which items reflect both specific dimensions (e.g., cognitive-motor activation) as well as a global overarching construct (instructional quality) can be considered a second source of construct-relevant psychometric multidimensionality (Reise et al., 2010; Morin et al., 2020). In this context, higher-order models can be distinguished from bifactor models. In higher-order models, the indicators reflect the orthogonally set first-order factors, which in turn reflect the second-order factor. Accordingly, the second-order factor has no direct effect on the indicators, but only indirectly via the first-order factors. In bifactor models, the higher-order global factor (G factor) directly influences the indicators [e.g., Reise et al., 2010; see also Schmid and Leiman (1957) transformation procedure (SLP)]. In the context of ICM-CFA, this would mean that all item loadings of the G factor as well as of the specific factors (S factors) would be freely estimated, with the factors set orthogonally as in higher-order models (Morin et al., 2020). The variance in the bifactor model can thus be divided into a global component of the shared variance of all indicators, additional specific components of the shared covariance of a subset of specific items, and a measurement error. Accordingly, the restrictive assumption of higher-order models that the association between indicators and higher-order factors are fully mediated by the first-order factors leads to a significantly poorer fit to the data than in bifactor models (e.g., Reise, 2012; Gignac, 2016). These observations strongly support the use of bifactor models as the preferred approach for accurately separating the variance in indicators, distinguishing between what can be attributed solely to overarching factors and what is specific to individual constructs (Morin et al., 2020).

In the context of the study of instructional quality, it can be assumed that there is both a hierarchically ordered construct and that the S-factors have a conceptual overlap. In order to account for these features, it is appropriate to integrate a combination of ESEM and bifactor modeling into one model, which has recently become possible through the development of the bifactor ESEM framework (Morin et al., 2020). Thus, it becomes possible to address for potential cross loadings and to investigate the explanatory power of the S-factors as well as the G-factor simultaneously. This aspect holds significant importance because research indicates that neglecting both layers in predictive models, assuming their coexistence, poses the risk of overlooking valuable insights into the distinctive impact of each S-Factor beyond the G-Factor. Neglecting to evaluate global factors within the structural model could lead to an overestimation of the specific factors’ influence and result in an incomplete understanding of the general factor (Alamer, 2022).

## 2 Research questions and hypothesis

We investigate the transferability and adaptation of an extended model of the three basic dimensions as a parsimonious model of instructional quality for PE. In doing so, the theoretical foundations of the previous sections lead us to the following research questions and hypotheses:

1. To what extent does the five-factor model of instructional quality in physical education represent the model to be favored with the best fit to the data? We hypothesize that a latent factor model with five individual- and class-level dimensions will provide the best fit to the data (H1).
2. Given different individual-level modeling approaches, to what extent can the factor structure of instructional quality be described? We assume the B-ESEM to yield the best fit to the data and that the model can give us otherwise inaccessible, valuable evidence about the internal structure of the data (H2).
3. To what extent can substantial cross-loadings on the untargeted factors be identified? We assume that, due to the high inter-factor correlations to be assumed, there are significant cross-loadings of non-targeted factors between cognitive-motor activation, motivational-emotional support and adjusted and blanked cognitive-motor support. However, the highest factor loadings in each case correspond to the target factors (H3).
4. To what extent are the items of classroom management be reflected in the G-factor? Based on theoretical rationales and empirical evidence, we assume that our parsimonious conceptualization of classroom management has considerably smaller factor loadings with respect to the G-factor (H4).

## 3 Materials and methods

### 3.1 Procedure and participants

Data stems from the anonymzed study (anonymized authors) has to be adapted in: “EPiC-PE study” and “Messmer et al. (2022)” which aims to investigate the effects of professional competencies of PE teachers on instructional quality and students’ outcomes. We focus in particular on the second measurement point, at which instructional quality was surveyed, referring to a 12-lesson teaching series. For the recruitment of the participants, secondary schools in several German-speaking cantons of Switzerland were contacted. Data collection took place between October 2021 and April 2022. The completion time of the whole survey section took 15–20 min at each measurement point and was complemented by a knowledge test. Beforehand, the students received a short explanation from their teacher, who was trained for this purpose by means of a standardized written explanation. Parents were informed prior that participation was voluntary and were required to sign an informed consent form. Students were also informed that participation was voluntary. No incentives were given for participation. The teachers had to complete their own questionnaire and were present during the entire assessment. The total sample consists of 72 different classes and 1,047 students. The average class size for the sample is 14.5 students per class. The average age of students drawn from grades 9 to 11 is 14.5 years (SD = 1.6). Forty-seven percent of the subjects were female.

### 3.2 Measures

In the context of the parsimonious modeling approach, the operationalization of instructional quality is based on a hybrid concept that combines generic with subject-specific quality characteristics (e.g., Kyriakides et al., 2018; Praetorius and Charalambous, 2018). Due to the minor adjustments for classroom management (CM) compared to other subjects, as well as the focus of the present study to present a parsimonious instrument, the focus was exclusively on low-level disruption. It is likely that variables of a broader understanding of CM (e.g., transition management and monitoring) would ultimately manifest in low-level disruption in the classroom, which in turn should allow for more effective learning time. Although different specifications could have been made for PE (e.g., safety aspect, use of materials), this parsimonious operationalization allows for easier integration into more complex models (e.g., effectiveness analyses). The items were adapted from the DESI and IGLU study (Bos et al., 2005; Wagner et al., 2009).

Due to the broad existing evidence regarding motivational and emotional processes in PE, an attempt was made to combine as many relevant aspects as possible in a consistent and, as it were, parsimonious scale of motivational-emotional support (MES). Accordingly, the items reflect both autonomy and competence support of the SDT as well as the teacher-student relationship in terms of relatedness. Furthermore, a motivating teaching style, a positive feedback approach, and an individual reference norm orientation were integrated (see section 1.3.2). The items were adapted from the COACTIV, DESI, and IGLU study as well as the General Self-Efficacy Scale (Schwarzer and Jerusalem, 1999; Bos et al., 2005; Baumert et al., 2009; Wagner et al., 2009).

Adjusted cognitive-motor support (ACMS) contains indicators that reflect modeling, explaining and highlighting in the context of augmented feedback. On the one hand, it refers to the correctness of the exercise execution; on the other hand, it explicitly focuses on the identification and correction of errors in movement execution. Therefore, it integrates both informational and corrective feedback. Blanket cognitive-motor support (BCMS) contains items that focus in particular on developmentally sequenced activities as well as the pre-movement emphasis on important movement elements and goals. The focus is on the role of teacher guidance of students in complex learning environments, aiming to reduce complexity as well as clear outline of the objectives of the exercises.

Finally, the dimension of cognitive-motor activation (CMA) includes challenging tasks, exploration of students’ movement actions, and metacognitive learning. It is assumed that “prior knowledge” (in PE rather the prerequisite of motor competence) manifests itself in the adequacy of the level of challenge, which is reflected in the items. One difficulty lay in the question of whether higher order thinking, analogous to other subjects, is beneficial to learning success in PE or rather inhibits the automation of movements. Following the generic model of the three basic dimensions, higher-order thinking is integrated into the scale, but the impact is an open question that should be addressed in subsequent studies of prognostic evidence. Items for both dimensions of cognitive-motor support and CMA has been adapted from Herrmann (2019).

## 4 Analysis

First, we conducted single-level ICM-CFA and MCFA that was specified doubly latent accordingly to the approach of Marsh et al. (2009). We compared the hypothesized five-factor structure with the four-factor structure (ACMS and BCMS represent one dimension) and other alternative models of the common integration of facets of cognitive support into the three basic dimensions (see Section 1.2). After a potentially different factor structure appeared on the different levels, we compared a model with five-factors at the individual-level and four-factors at the class level with the alternative models.

**Figure 1:**
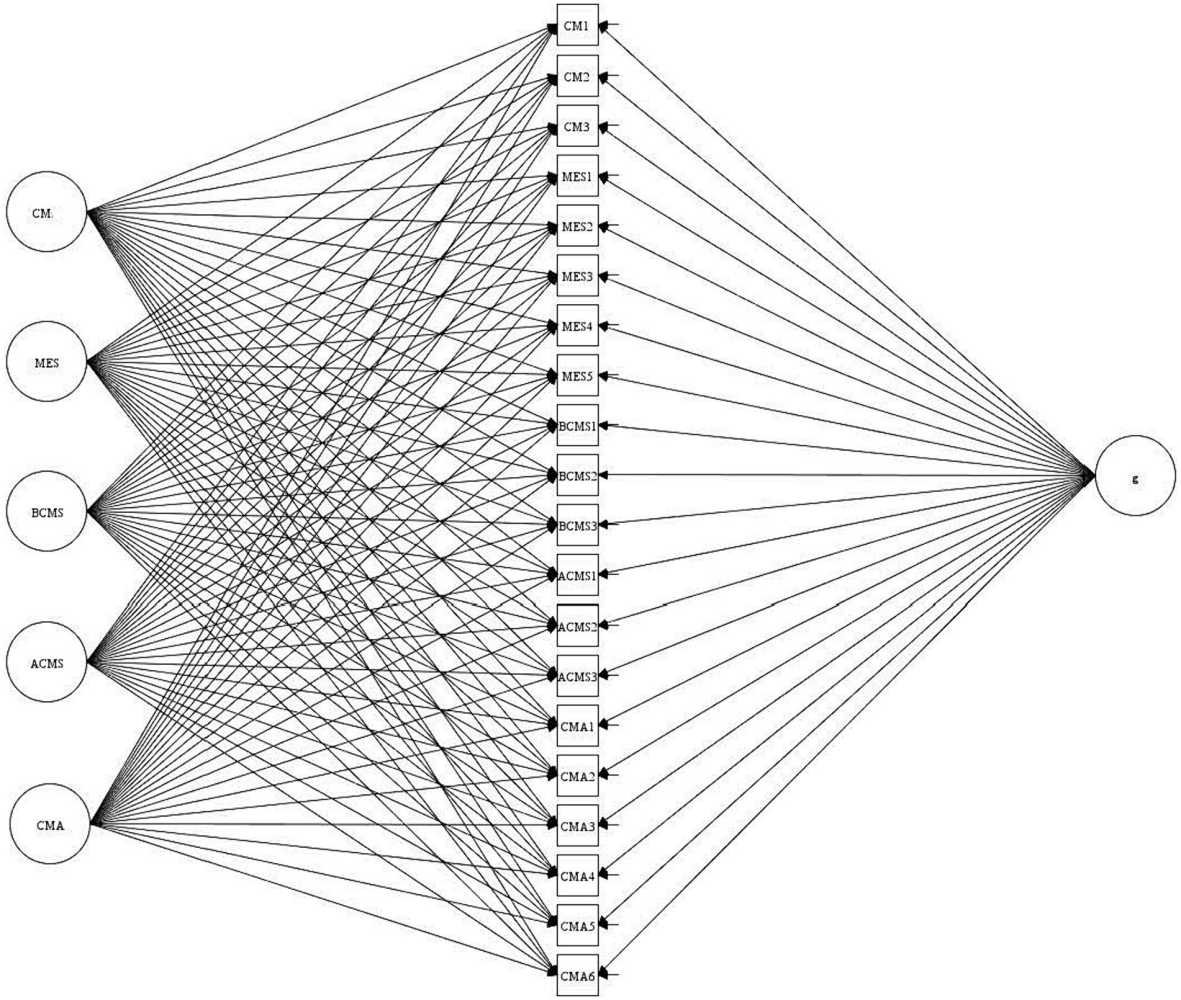
B-ESEM solution of the measurement model. CM, Classroom management; MES, Motivational-emotional support; BCMS, Blanket cognitive-motor support; ACMS, Adjusted cognitive-motor support; CMA, Cognitive-motor activation.

Regarding the ESEM and B-ESEM, the first step of a sequential analysis strategy was presented in the theory section as a rationale for the usefulness of assuming a hierarchically ordered construct that has conceptual overlap in dimensions (Morin et al., 2020). Following Morin et al. (2016), the second step was to compare ICM-CFA and ESEM to test for the presence of construct-relevant psychometric multidimensionality. In this step, ESEM should show a better fit to the data, inter-factor correlations should decrease, and low to moderate cross-loadings should emerge. Larger cross-loadings should be able to be explained well and the factors should be well defined. The third step consists of a comparison of the model to be favored (CFA or ESEM) with a bifactor solution (B-CFA or B-ESEM). An improvement of the model fit as well as a well-defined G-factor can be considered as evaluation criteria. The S-factors should be at least partially well defined, although it is not necessarily considered critical in bifactor models for all S-factors, as these serve as controls of residual specifications shared between a subset of indicators (Morin et al., 2020). ESEM and B-ESEM were conducted with oblique target rotation (Figure 1).

All models were estimated using Mplus 8.7 (Muthén and Muthén, 2017) with robust Maximum Likelihood estimation (MLR), which is robust against non-normality of item responses. Despite the categorical variables, we preferred MLR estimation over weighted least squares means and variance adjusted (WLSMV) estimation, following the practice of Aguado et al. (2015) and Scherer et al. (2016). In this context we specify at least four response options on a frequency scale, we can use the “missing at random” (Asparouhov and Muthén, 2010) handling of missing data, and we follow the recommendation of Marsh et al. (2009) regarding the use of MLR estimation in the application of ESEM. Goodness-of-fit was assessed using the absolute fit indices, adhering to conventional cutoff values from Hu and Bentler (1999): standardized root mean square residual (SRMR) ≤ 0.08; root mean square error of approximation (RMSEA) ≤ 0.06; comparative fit index (CFI) and tucker-lewis index (TLI) ≥ 0.95 as well as χ^2^/df-Ratio (Wheaton et al., 1977). Additionally, lower Akaike Information Criteria (AIC), Bayesian Information Criteria (BIC), and chi-square difference testing using the Satorra-Bentler scaled chi-square indicated favorable models (Morin et al., 2016). The COMPLEX function of Mplus (Asparouhov, 2005) was used for all individual-level models to estimate goodness-of-fit and standard errors robust to the nested data structure. The proportion of missing values per item was between 0.0 and 1.9%. Missing values were addressed using the full information maximum likelihood estimator (FIML).

## 5 Results

### 5.1 Descriptives

All Items with descriptives can be found in the Appendix. Intraclass correlation coefficients (ICCs) as well as design effects indicated substantial dependence of clustering of the data within classes. In this context, ICC1 values higher than 0.05 indicate meaningful correlations of variables between and within. ICC2 values higher than 0.60 indicate a meaningful aggregation of the individual-level data on the class level (Bliese, 2000; Chen et al., 2005). Only CMA showed ICC1 values that were only slightly above the cut-off of 0.05. The ICC2 values were below 0.60 and also the design effects were below 2.0. Accordingly, the reliability of the scale at the class level can be described as insufficient. Table 1 shows an overview of the descriptives.

**Table 1:** Descriptives of instructional quality dimensions.

		Item example (1 = not true at all; 4 = exactly true)						
Factor	Items	(“In our physical education class…”; “Our physical education teacher…”).	*M*	*SD*	α	ω	ICC1	ICC2
Classroom management	3	There are many disruptions by students. (*r*)	2.84	0.72	0.84	0.84	0.15–0.21	≥0.70
Motivational-emotional support	5	Often makes physical education lessons really exciting.	3.02	0.64	0.81	0.81	0.12–0.18	≥0.66
Blanket cognitive-motor support	3	Gradually introduces the exercises step by step.	3.21	0.63	0.82	0.82	0.13–0.14	≥0.67
Adjusted cognitive-motor support	3	Shows us errors in the execution of exercises.	3.23	0.62	0.86	0.86	0.13–0.15	≥0.68
Cognitive-motor activation	6	Makes me think about how I should execute the exercises.	2.82	0.63	0.87	0.87	0.06–0.08	≥0.47

### 5.2 Results of the ICM-CFA

To address Research Question 1, we first examined the different alternative models at the individual level using ICM-CFA. We examined the extent to which a unidimensional factor had an acceptable model fit, assuming that we were measuring the superior construct of instructional quality. This model had a poor model fit (Table 2) and was therefore rejected. The next step was to examine the possible alternative models that integrated cognitive-motor support within the other basic dimensions. Integration into CM was not considered because the parsimonious operationalization did not suggest a meaningful integration in terms of content. The integration into CMA showed a poor model fit, whereas the integration into MES showed an acceptable, if not good model fit. Next, the four-factor model of an integrative dimension of BCMS and ACMS was tested. This solution resulted in a significantly better model fit compared to the alternative models. Finally, the postulated five-factor model was tested, in which BCMS and ACMS represent independent dimensions. The model shows a significant improvement of the model fit with respect to the Satorra-Bentler Scaled Chi-Square difference test. RMSEA, CFI, and TLI each improved by 0.01, whereas SRMR remained the same. AIC and BIC also indicate a preference for the five-factor solution. In summary, the five-factor model represents the model to be favored, following common cut-off values of model comparison (Table 2).

**Table 2:** Fit statistics of ICM-CFA measurement models.

Model	Specifics	χ^2^	df	χ^2^/df	RMSEA	CFI	TLI	SRMR	AIC	BIC	ΔCFI	ΔRMSEA	TRd	Δdf	*p*
1	CM, MES, ACMS, BCMS, CMA	371.89^**^	160	**2.32**	**0.03**	**0.97**	**0.97**	**0.04**	**38,506**	**38,853**					
2	CM, MES, CMS, CMA	440.76^**^	164	2.69	0.04	0.96	0.96	**0.04**	38,595	38,922	0.01	0.01	59.11	4	0.00
3	CM, MES, CMA+ CMS	728.70^**^	167	4.36	0.06	0.93	0.92	0.05	38,986	39,297	0.04	0.03	306.9	7	0.00
4	CM, MES + CMS, CMA	1433.10^**^	167	8.58	0.09	0.84	0.82	0.07	39,932	40,245	0.13	0.06	1103.28	7	0.00
5	General	2609.73^**^	170	15.35	0.12	0.7	0.66	0.10	41,464	41,761	0.27	0.09	2299.58	10	0.00

CM, Classroom management; MES, Motivational-emotional support; CMS, Cognitive motor support (compound); ACMS, Adjusted cognitive motor support; BCMS, Blanket cognitive motor support; CMA, Cognitive motor activation; ^**^p < 0.01. Bold values indicate the best model fit.

### 5.3 Results of the MCFA

In a next step, the factor structure was tested simultaneously at individual and class level using MCFA (Table 3). The procedure as well as the results regarding the unidimensional model and the integration into the three-factor solutions on individual and class level are largely congruent with the ICM-CFA. However, when comparing the four- and five-factor solution, no improvement in the SRMR between could be demonstrated, while the fit indices at the individual level indicated a better fit to the data. Accordingly, Model 1b was specified, which tests a five-factor structure at the individual level and a differential four-factor structure at the class level. Model 1b did not show a worse model fit and even showed better values for the AIC and BIC than Model 1a. In addition, there was an almost perfect inter-factor correlation at the class level between BCMS and ACMS (see Table 4). Table 5 presents the inter-factor correlations at the class level with respect to the four-factor solution, with the two highest inter-factor correlation at 0.91 in a common range for the class level. The overall correlational pattern is in line with expectations, being higher between conceptual closer dimensions at both levels. The different factor structure on the two levels is not unusual in the context of MCFA, as multilevel models often tend to show a simpler factor structure at the class level compared to the individual level (Dedrick and Greenbaum, 2011). All factor loadings were found to be statistically significant. On the student level, standardized loadings for all items ranged from 0.47 to 0.81, while on the class level, the range was from 0.59 to 1.00.

**Table 3:** Fit statistics of the MCFA.

Model	Specifics	χ^2^	df	χ^2^/df	RMSEA	CFI	TLI	SRMR within	SRMR between	AIC	BIC	ΔCFI	ΔRMSEA	TRd	Δdf	*p*
1a (5/5)^*^	CM, MES, ACMS, BCMS, CMA	756.20^**^	335	2.26	**0.03**	**0.96**	**0.96**	**0.04**	**0.10**	38,136	38,656					
1b (5/4)^*^	Individual: CM, MES, ACMS, BCMS, CMA	754.41^**^	339	**2.23**	**0.03**	**0.96**	**0.96**	**0.04**	**0.10**	**36,185**	**36,666**	0	0	0.63	4	0.96
	Class: CM, MES, CMS, CMA															
2 (4/4)^*^	CM, MES, CMS, CMA	854.86^**^	344	2.49	0.04	0.95	0.95	**0.05**	**0.10**	38,215	38,691	0.01	0.01	75	9	0.00
3 (3/3)^*^	CM, MES, CMA + S	1270.91^**^	351	3.62	0.05	0.91	0.90	0.05	0.12	38,592	39,033	0.05	0.01	364	17	0.00
4 (3/3)^*^	CM, MES + CMS, CMA	2300.67^**^	351	6.55	0.07	0.81	0.79	0.07	0.19	39,530	39,970	0.16	0.04	1,142	16	0.00
5 (1/1)^*^	General	3791.53^**^	359	10.56	0.10	0.66	0.65	0.10	0.25	40,915	41,316	0.3	0.06	2,454	24	0.00

CM, Classroom management; MES, Motivational-emotional support; CMS, Cognitive motor support (compound); ACMS, Adjusted cognitive motor support; BCMS, Blanket cognitive motor support; CMA, Cognitive motor activation; ^*^Number of factors at individual/class level; ^**^p < 0.01.

**Table 4:** Inter-factor correlations of the five-factor solution at both levels.

Factor	CM	MES	BCMS	ACMS	CMA
CM	1	0.07	0.05	0.01	0.05
MES	0.43^**^	1	0.77^**^	0.71^**^	0.59^**^
BCMS	0.53^**^	0.93^**^	1	**0.87** ^**^	0.60^**^
ACMS	0.51^**^	0.90^**^	0.998^**^	1	0.60^**^
CMA	0.05	0.88^**^	0.67^**^	0.64^**^	1

Inter-factor correlations at individual level above the diagonal; class level below the diagonal; highest expected inter-factor correlations in bold; decision-supporting values of the undifferentiated factor structure at class level are underlined; ^**^p < 0.01.

**Table 5:** Inter-factor correlations of the four-factor solution at class level.

FaCtor	CM	MES	CMS	CMA
CM	1			
MES	0.43^**^	1		
CMS	0.52^**^	0.91^**^	1	
CMA	0.05	0.88^**^	0.65^**^	1

^**^p < 0.01.

### 5.4 Results of the ESEM and B-ESEM

To address research questions 2, 3, and 4 in a next step, we specified an ESEM and B-ESEM solution. The sequential procedure first consisted of testing the presence of construct-relevant psychometric multidimensionality using ESEM. As Table 6 shows, the ESEM solution had an excellent model fit and outperformed the ICM-CFA (ΔCFI = +0.02, ΔTLI = +0.02, ΔSRMR = −0.03, AIC, BIC). Furthermore, the inter-factor correlations decreased substantially (Table 7). As for the MCFA, the correlational pattern is in line with expectations, being higher between conceptual closer dimensions. Also in line with expectations are the low inter-factor correlations with CM, which already serves as an indication with regard to research question 4.

**Table 6:** Comparison of the fit statistics of the ICM-CFA, ESEM, and B-ESEM.

Model	Specifics	χ^2^	df	χ^2^/df	RMSEA	CFI	TLI	SRMR	AIC	BIC	ΔCFI	ΔRMSEA	TRd	Δdf	*p*
1	B-ESEM	100.88	85	**1.19**	**0.01**	**1**	**1**	**0.01**	**38,282**	**38,540**					
2	ESEM	163.76^**^	100	1.64	0.03	0.99	0.99	**0.01**	38,347	38,578	0.01	0.02	52.31	15	0.00
3	ICM-CFA	371.89^**^	160	2.32	0.03	0.97	0.97	0.04	38,506	38,630	0.03	0.02	263.5	75	0.00

^**^p < 0.01.

**Table 7:** Factor loadings of the three measurement approaches and inter-factor correlations of ICM-CFA and ESEM solution.

Items	CFA	ESEM					Bifactor ESEM					
		CM	MES	BCMS	ACMS	CMA		CM	MES	BCMS	ACMS	CMA
	𝛽	𝛽	𝛽	𝛽	𝛽	𝛽	G-𝛽	S-𝛽	S-𝛽	S-𝛽	S-𝛽	S-𝛽
CM1	**0.82** ^**^	**0.82** ^**^					**0.10** ^*^	**0.81** ^**^				
CM2	**0.82** ^**^	**0.82** ^**^					**0.11** ^**^	**0.81** ^**^				
CM3	**0.75** ^**^	**0.74** ^**^					**0.16** ^**^	**0.73** ^**^				
												
MES1	**0.72** ^**^		**0.64** ^**^			0.12^**^	**0.61** ^**^		**0.35** ^**^			0.10^**^
MES2	**0.72** ^**^		**0.83** ^**^	−0.11^*^			**0.58** ^**^		**0.50** ^**^			
MES3	**0.77** ^**^		**0.90** ^**^				**0.63** ^**^		**0.51** ^**^			
MES4	**0.68** ^**^		**0.58** ^**^				**0.60** ^**^		**0.30** ^**^			
MES5	**0.67** ^**^		**0.38** ^**^	0.16^*^		0.16^**^	**0.62** ^**^		**0.18** ^**^			0.10^*^
												
BCMS1	**0.77** ^**^		0.10^*^	**0.66** ^**^			**0.77** ^**^			**0.19** ^**^		
BCMS2	**0.79** ^**^		0.10^**^	**0.48** ^**^	0.29^**^		**0.74** ^**^			**0.19** ^**^	0.13^**^	−0.10^**^
BCMS3	**0.78** ^**^			**0.58** ^**^	0.12	0.12^**^	**0.76** ^**^			**0.20** ^**^		
												
ACMS1	**0.83** ^**^			0.34^**^	**0.52** ^**^		**0.78** ^**^				**0.24** ^**^	
ACMS2	**0.79** ^**^			−0.14^*^	**0.91** ^**^		**0.68** ^**^				**0.52** ^**^	
ACMS3	**0.84** ^**^			0.14^*^	**0.66** ^**^		**0.74** ^**^				**0.36** ^**^	
												
CMA1	**0.74** ^**^					**0.77** ^**^	**0.49** ^**^			−0.21^*^		**0.57** ^**^
CMA 2	**0.77** ^**^					**0.76** ^**^	**0.55** ^**^			−0.27^*^		**0.56** ^**^
CMA 3	**0.80** ^**^					**0.82** ^**^	**0.50** ^**^			0.13^*^		**0.63** ^**^
CMA 4	**0.78** ^**^				0.11	**0.73** ^**^	**0.50** ^**^			0.16^**^	0.12^**^	**0.60** ^**^
CMA 5	**0.79** ^**^					**0.81** ^**^	**0.49** ^**^			0.11^*^		**0.62** ^**^
CMA 6	**0.48** ^**^		0.19^**^	0.24^**^	−0.10	**0.29** ^**^	**0.50** ^**^				−0.11^*^	**0.18** ^**^
	Inter-factor correlations for ICM-CFA (above the diagonal) and ESEM (below the diagonal)											
Factor	CM		MES		BCMS		ACMS			CMA		
CM	1		0.09		0.06		0.01			0.04		
MES	0.16^**^		1		0.76^**^		0.70^**^			0.60^**^		
BCMS	0.11^*^		0.69^**^		1		**0.87** ^**^			0.62^**^		
ACMS	0.13^**^		0.67^**^		**0.72** ^**^		1			0.62^**^		
CMA	0.04		0.58^**^		0.51^**^		0.52^**^			1		

Target loadings are in bold; loadings below 0.1 are not shown; highest expected inter-factor correlations in bold; ^*^p < 0.05; ^**^p < 0.01.

Regarding the ESEM factor loadings (Table 7), target loadings above 0.50 are considered completely satisfactory following Morin et al. (2020). Target loadings below 0.30 question the adequacy of the indicator. The target loadings of the ESEM solution are in an acceptable range except for item CM6 (0.29), and even in a completely satisfactory range except for item BCMS2 (0.48). All cross loadings are in a negligible range (<0.40), whereby individual attention should be paid to both the justifiability of the content and the relative height to the target loading. In principle, it should be noted that cross-loadings only reflect the construct-relevant association between an indicator and a non-target factor, so that higher cross-loadings may be tolerated if they make theoretical sense (Morin et al., 2020). In line with expectations, cross-loadings worth mentioning occur for item CM6 as well as for the theoretical aligned BCMS and ACMS items. Importantly, these cross-loadings may suggest that an unmodelled G-factor might be present (Morin et al., 2020). Furthermore, since ESEM is supported by the improvement of the model fit, the reduced inter-factor correlations, low to moderate cross-loadings and well-defined target factors, a B-ESEM solution was specified in a next step.

Regarding research question 2, Table 6 shows that the B-ESEM solution had an even better model fit than the ESEM solution (e.g., ΔCFI = +0.01, ΔTLI = +0.01, ΔRMSEA = −0.02). Furthermore, the B-ESEM solution shows a well-defined G-factor and resulted in a non-significant chi square value (*p* = 0.12), suggesting that it is the only model with exact model fit to the data. However, as we assumed for research question 4, items assigned to CM showed only small loadings on the G-factor (λ = 0.09–0.13), but high loadings on the S-factor (λ = 0.73–0.81). The other target factors can also be described as predominantly well defined. With regard to research question 3, the highest factor loadings in each case correspond to the target factors. Interestingly, item CM6 also has significant loadings on the G-factor and the S-factor. The cross-loadings observed in the ESEM thus appear to be mainly explained by the shared G-factor.

## 6 Discussion

The main goal of the present study was to transfer and extend a popular model of generic research on instructional quality to PE. As part of a multi-step procedure, the factor structure was examined. Further studies are indicated in the future for in-depth analysis on prognostic validity and prerequisites like teacher’s professional competencies or continuing professional development (Tannehill et al., 2021; Büchel et al., 2023). Various subject-specific adaptations and additions were made and grounded against the background of substantial theoretical and empirical evidence. ICM-CFA and MCFA were applied to examine the factorial structure of instructional quality in PE. Within the ICM-CFA, it could be shown that the postulated model with five factors showed both a good model fit and the best model fit in comparison to the alternative models. Regarding the MCFA, the ICC1 and ICC2 values were first calculated as a measure of the degree of dependency of the data within classes. The ICC1 values of CMA were found to be rather low, whereas CM showed the highest values. MES, BCMS, and ACMS had similar ICC1 and ICC2 values. These findings are largely in line with expectations and can be justified on the basis of the degree of inference (e.g., Wisniewski et al., 2020). The low ICC1 values for CMA tend to be at the lower end of the values reported in studies of other subjects (e.g., Fauth et al., 2014; Wisniewski et al., 2020). One possible explanation for the lower ICC1 values of the CMA could lie in the item reference on the individual student, whereas the other factors are more strongly aimed at general teaching or the teacher (Fauth et al., 2020). Accordingly, the differences in the ICC1 values could be explained less by the construct but rather by the item reference. Therefore, consideration of the item reference seems to be a worthwhile investigation in further studies. Previous research has found different combinations of item references between and within constructs, which appear to be associated with the ICC1 values (Holzberger et al., 2013; Fauth et al., 2014). This differentiated psychometric consideration, which is relatively new in research on instructional quality, is also a potentially important approach for explaining inconsistent findings with regard to the predictive validity of students’ perceptions as well as with regard to the low level of agreement with other data sources such as external observations. With the exception of CMA (≥0.47), the ICC2 values showed satisfactory reliability of the aggregated class mean values. For the focus of the analysis at class level, a careful adjustment of CMA appears to be indicated.

With regard to the inter-factor correlations, the results are largely consistent with other studies (Kane et al., 2014; Röhl and Rollett, 2021). Accordingly, with the exception of CM, high inter-factor correlations can be reported between the dimensions at both individual and class level. With regard to CM, it is advisable to take a closer look at the specific operationalization. In studies that have operationalized CM in a broader sense or with a focus on other facets (e.g., Wagner et al., 2013; Wisniewski et al., 2020), higher inter-factor correlations can be observed, whereas studies with a focus on low-disruptive behavior tend to report similar findings (e.g., Fauth et al., 2014; Kleickmann et al., 2020). No significant inter-factor correlation was found between CM and cognitive-motor activation at class level either, whereas substantial inter-factor correlations were found between CM with cognitive-motor support and MES (Table 5). Finally, MES in particular shows high inter-factor correlations. The question of the influence of an affective overall attitude in the sense of perceived “communion” can be cited here in particular as a question and at the same time as a possible explanation (Kuhfeld, 2016; Wallace et al., 2016; Röhl and Rollett, 2021).

While the postulated model with five factors for the individual level showed the best model fit, a less differentiated factor structure was evident at the class level. Against this background, no improvement in the model fit resulted by differentiating the two components of cognitive-motor support, AIC, and BIC even pointed out the preference for the four-factor model. In contrast, a structure with four factors at the individual level showed a poorer model fit (ΔCFI = −0.01, ΔRMSEA = +0.01, AIC, BIC). Therefore, as a result of model fit, theoretical stringency in terms of the conceptually adjacent dimensions and the principle of parsimony, Model 1b represented the adopted model. From a theoretical point of view, no different interpretation was postulated at the two levels of analysis, although a simpler factor structure at the higher level can be described as a common phenomenon (e.g., Dedrick and Greenbaum, 2011). Nevertheless, it could also be shown for the class level that an extension of the model of the three basic dimensions for PE by a cognitive-motor support dimension represents a theoretically as well as empirically meaningful addition.

In a further step, the individual level was examined using more complex methodological techniques (ESEM, B-ESEM). Overall, it was shown that all three modeling approaches (ICM-CFA, MCFA, ESEM, and B-ESEM) exhibited a good model fit, with the more complex modeling approaches outperforming the ICM-CFA. Basically, the findings show that the students’ perception is able to distinguish between the different factors of instructional quality for PE. The findings of the ESEM show significant cross-loadings, which reflect the conceptual overlap of the constructs. In the context of arguments for convergent and discriminant validity, it should first be emphasized that the cross-loadings emerge in line with expectations between theoretically more strongly associated constructs. In particular, significant cross-loadings of the BCMS and ACMS items appear. Regarding research question 3, the target loadings consistently represent the highest factor loadings and the factors can be described as well defined. Only item CM6 has a target loading <0.30 and higher cross-loadings. The item wording (“Our PE teacher gives us different exercise tasks, depending on our ability”) does indeed differ from the other items, which focus more on higher-order thinking, exploration of students’ movement actions, and metacognitive learning (see Appendix). Even if the assignment to CMA can be justified within the framework of the generic model of the three basic dimensions, we consider the connection to motivational and emotional processes as well as to ACMS and BCMS in the sense of cognitive load to be just as viable, which is congruent with the notion of the connection between the student-teacher relationship and feedback in PE (Zhou et al., 2021). As expected, the inter-factor correlations of the ESEM solution are lower than those of the ICM-CFA, with the exception of CM. This can be considered particularly significant if the latent variables are to be used for predictions, as in this case in further studies, and unnecessary multicoloniality would be introduced (Asparouhov et al., 2015; Howard et al., 2016). In the context of research on instructional quality, this can be considered problematic due to the conceptual overlap and the correspondingly high inter-factor correlations.

With regard to the B-ESEM, CM with low factor loadings on the G-factor was particularly striking. This was expected both in the context of the lower inter-factor correlations of CM with the other factors, but especially in the context of initial evidence regarding B-ESEM in the context of the three basic dimensions (Scherer et al., 2016). In this context, the inter-factor correlations of all modeling approaches underline the assumption of conceptual closeness of the other dimensions compared to CM, showing that stronger inter-factor correlations occur between conceptually adjacent factors and lower inter-factor correlations between conceptually distal factors. Otherwise, the G-factor is well defined for all other dimensions and supports the assumption of the presence of a superordinate factor. The loadings on the S-factors all show significant target loadings, indicating that they can explain variance beyond the G-factor.

Overall, the findings of our study provide strong support for the factorial structure of the measurement model in question. In this context, a foundation of instructional quality from a hybrid perspective, which integrates generic and subject-specific approaches, appears to be a promising direction. Assuming conceptually overlapping dimensions and a general factor of instructional quality, we were able to use the more complex modeling approaches (ESEM, B-ESEM) to address both the cross-loadings among items and factors and to disentangle the variance explained by the general factor and the specific factors. This seems particularly important in light of current challenges in research on instructional quality, as problems regarding factor mean differences and the relationship to other constructs can be addressed.

Nevertheless, different limitations of the measurement model can be identified. First, certain aspects could not be integrated. Especially, transferring evidence on focus of attention into the instrument can be understood as potentially important. Furthermore, in the context of the study’s focus on a parsimonious model, we had to make further limitations, such as the focus on disruptive behavior or verbal feedback, as the most common source of augmented feedback in PE. Second, it should be emphasized that there is an ongoing debate about the extent to which laboratory studies of motor learning can be transferred to everyday settings (e.g., Wolpert et al., 2011). Third, according to the literature on motor learning as well as models of PE, there may be contradictions where it cannot be conclusively assessed in which context which approach would be beneficial. For example, the discovery-based learning (DBL) model and high structuring of lessons are opposed to each other. Likewise, the methodological series of exercise model or the methodical games series model are not necessarily compatible with DBL. Against this background, it should be emphasized that different approaches should certainly be evaluated against the background of the objective of individual lessons. The measurement model presented can therefore only be understood in the context of overarching quality dimensions by reducing the complexity of teaching. Fourth, the greatest limitation is certainly the current lack of evidence regarding further arguments of validity like the effects on significant educational outcomes, which has to be addressed in further studies. The present study can therefore be seen as a first step, in the sense of a multi-step procedure, for arguments regarding the factorial validity of the instrument.

## Data availability statement

The datasets presented in this article are not readily available because there is a data embargo until the completion of dissertations associated with the project by January 31, 2025. Requests to access the datasets should be directed to felix.kruse@phsg.ch.

## Ethics statement

Ethical approval was not required for the studies involving humans because in accordance with national guidelines in connection with the collection of non-sensitive data, no ethics vote was required for the study. The active consent of the parents was obtained before the study was conducted. The studies were conducted in accordance with the local legislation and institutional requirements. The participants provided their written informed consent to participate in this study.

## Author contributions

FK: Formal analysis, Writing – review & editing, Writing – original draft. SB: Conceptualization, Writing – review & editing. CB: Conceptualization, Writing – review & editing.

## Appendix

**Table:**

item	Item formulation (“In our physical education class…”; “Our physical education teacher…”).	*M*	*SD*	*rit*	*ICC1*
Classroom management	often has to wait a long time until it is quiet. (r)	2.79	0.82	0.69	0.21
	there are many disruptions by students. (r)	2.74	0.82	0.69	0.21
	things are often chaotic. (r)	2.95	0.81	0.65	0.15
Motivational-emotional support	often makes physical education lessons really exciting.	2.95	0.83	0.65	0.15
	takes care of the students’ problems.	3.14	0.84	0.64	0.17
	tries to fulfill the wishes of the students as far as possible.	3.07	0.83	0.71	0.18
	praises students in particular when they have improved on their previous performance.	3.14	0.80	0.62	0.13
	it is recognized when I achieve something	3.00	0.78	0.58	0.12
Blanket cognitive-motor support	gradually introduces the exercises step by step.	3.19	0.76	0.68	0.14
	points out the important aspects of the exercises.	3.29	0.71	0.69	0.13
	makes the objectives of the exercises clear.	3.21	0.76	0.69	0.14
Adjusted cognitive-motor support	points out the correct execution of exercises.	3.25	0.68	0.72	0.14
	points out errors in the execution of exercises.	3.21	0.73	0.76	0.13
	gives us advice on how to improve exercise execution.	3.22	0.73	0.77	0.15
Cognitive-motor activation	encourages me to think through the exercises after performing them.	2.83	0.84	0.72	0.06
	encourages me to think about how well I did the exercises.	2.89	0.84	0.77	0.07
	encourages me to think about how I could learn new exercises.	2.81	0.83	0.76	0.06
	makes me think about how I should execute the exercises.	2.94	0.79	0.73	0.06
	makes me think about the benefits of the exercises for me.	2.88	0.82	0.74	0.08
	gives us different exercise tasks, depending on our ability.	2.61	0.93	0.50	0.08
